# Optical Detection of Degraded Therapeutic Proteins

**DOI:** 10.1038/s41598-018-23409-z

**Published:** 2018-03-23

**Authors:** William F. Herrington, Gajendra P. Singh, Di Wu, Paul W. Barone, William Hancock, Rajeev J. Ram

**Affiliations:** 1 Massachusetts Institute of Technology, Cambridge, Massachusetts 02139 United States; 2 Northeastern University, Boston, Massachusetts 02115 United States

## Abstract

The quality of therapeutic proteins such as hormones, subunit and conjugate vaccines, and antibodies is critical to the safety and efficacy of modern medicine. Identifying malformed proteins at the point-of-care can prevent adverse immune reactions in patients; this is of special concern when there is an insecure supply chain resulting in the delivery of degraded, or even counterfeit, drug product. Identification of degraded protein, for example human growth hormone, is demonstrated by applying automated anomaly detection algorithms. Detection of the degraded protein differs from previous applications of machine-learning and classification to spectral analysis: only example spectra of genuine, high-quality drug products are used to construct the classifier. The algorithm is tested on Raman spectra acquired on protein dilutions typical of formulated drug product and at sample volumes of 25 µL, below the typical overfill (waste) volumes present in vials of injectable drug product. The algorithm is demonstrated to correctly classify anomalous recombinant human growth hormone (rhGH) with 92% sensitivity and 98% specificity even when the algorithm has only previously encountered high-quality drug product.

## Introduction

The use of therapeutic proteins has become common practice in medicine and their success has stimulated significant investment towards the discovery and manufacture of a wide range of biopharmaceuticals. A central challenge of protein therapies is the potential to trigger antibody formation. Triggering such an immune response has consequences for both the efficacy of the therapy and the safety of the patient^1,2^. Several factors have been correlated to immunogenicity: these include impurities, such as protein aggregates^3^, differences in glycosylation, deamidation or oxidation^4,5^, and protein misfolding^6^.

Rapid quality assessment of therapeutic proteins is essential when there is the possibility of an insecure supply chain – resulting in the shipment of degraded proteins or in the sale of counterfeit products – or when there is a need to closely monitor and control biopharmaceutical manufacturing. Formulated proteins require cold distribution and storage, a major limiting factor for treating populations in rural and developing areas. In India, concerns about spoilage necessitate preventative measures such as discarding vaccine vials that have been distributed to three vaccination clinic events, even if the vials remain unopened, resulting in a vaccine wastage rate of roughly 60%^7–10^. Despite these policies, a study looking for freeze events during distribution found that up to 76% of the vaccine vials tested showed evidence of spoilage due to freezing^11^. Point-of-care applications are especially challenging as a quality determination must be made rapidly on small sample volumes in clinical settings that may be resource constrained.

The above immunogenic factors all involve changes in the molecular characteristics of the protein that may be possible to measure at the point of care using Raman spectroscopy. Here we present a Raman instrument capable of measuring proteins in solution at dose relevant concentrations and with a sample volume small enough that it could be taken from the overfill volume used in vial packing of drug products^12^, and demonstrate a classification technique that is capable of identifying degraded material based on changes in the scattered Raman spectrum.

Raman spectroscopy, discovered in 1928 by C.V. Raman and K.S. Krishnan^13^, is commonly used in the analysis of biological materials^14^ and has been used for the verification of primary and secondary structure of proteins since 1958^15–17^. The published examples of protein Raman spectra have typically been reported for solid powders and for solutions with concentrations in excess of 5 mg/mL, higher than many formulated products. Previous work has been limited to high protein concentrations because of the small cross-sections for Raman scattering coupled with the potential for photo-degradation of the protein under high illumination. Traditionally, to detect proteins at lower, dose-relevant, concentrations has required a switch to Surface Enhanced Raman Scattering (SERS).

**Figure 1:**
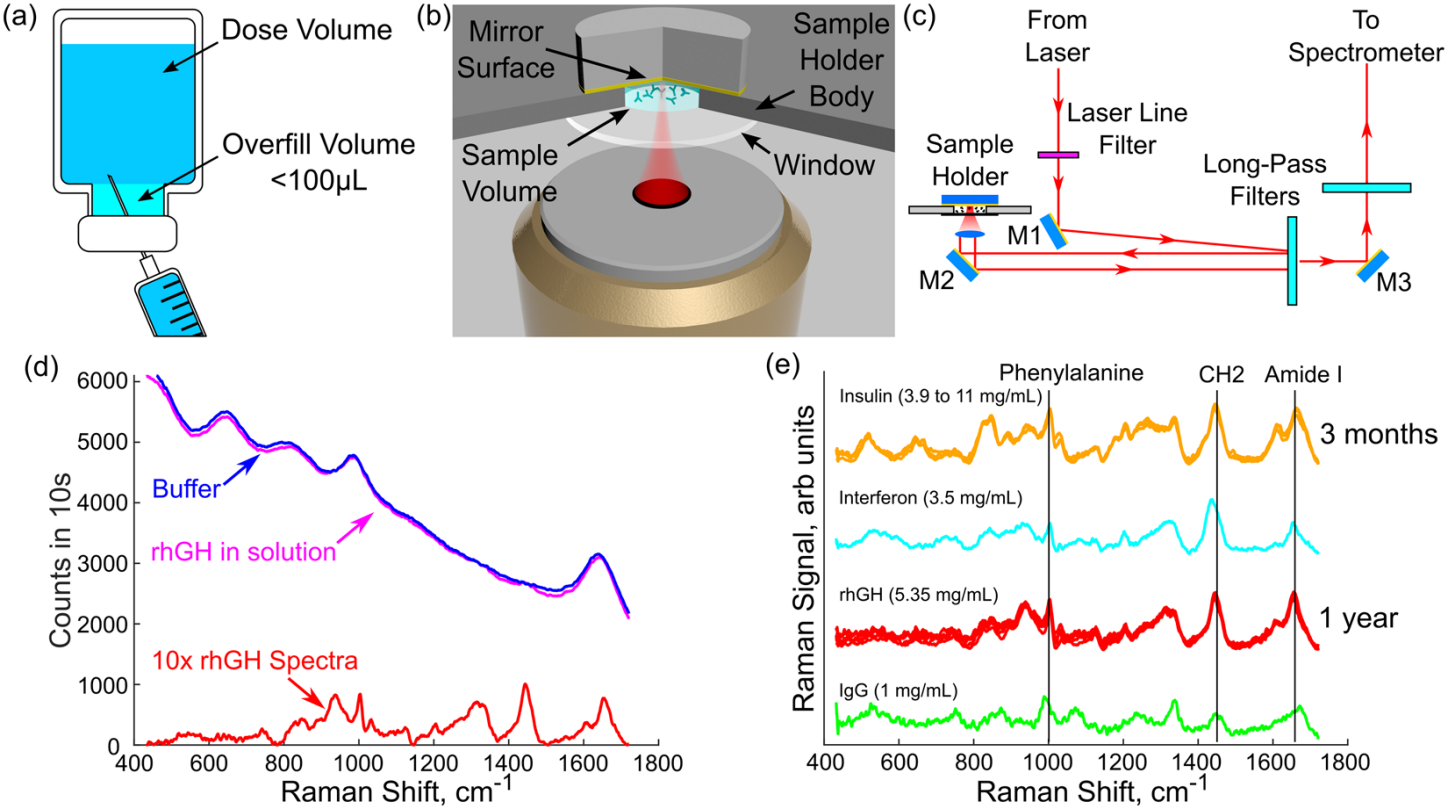
(**a**) The US pharmacopeia recommends that therapeutics supplied in a vial have some amount of overfill (excess) volume in the package to allow a complete dose to be drawn from the vial. Our sample holder, shown in (**b**) is designed with a sample volume of 25 µL, which is lower than the 100 μL overfill recommended for any drug product delivered in a vial 0.5 mL or greater. The total volume is kept low by recognizing that the volume of sample directly illuminated by the excitation light will produce most of the collected Raman signal. The depth of the sample holder was set so that the microscope objective used in this system, a Nikon Plan Fluorite 40 × 0.75NA objective, could focus on the mirror at the back of the sample holder. A schematic diagram of the Raman system beyond the sample holder is shown in (**c**). The raw signal for a protein in a buffer, the buffer, and the reconstructed protein spectrum (magnified 10×) is shown in (**d**). For the concentrations and samples examined here, the Raman signal has significantly lower intensity than the raw signal requiring careful background subtraction to retrieve the protein Raman signal. (**e**) Protein spectra from the four proteins examined during this project. To gauge the repeatability of the system several spectra were taken of rhGH over a year long period at a fixed concentration and spectra of Insulin over a three month period across a range of concentrations as indicated in (**e**). All spectra are plotted after normalization.

Surface Enhanced Raman Scattering is a technique where the protein is adsorbed to a metallic substrate, resulting in resonance enhancement of the excitation field^18–20^. The resonance enhancement enables detection down to the single molecule level^20^. The SERS signal is related to the constituent amino acids and amide backbone of the protein^21,22^, but the spectrum differs from the spectra of the protein in solution due to the fact that the SERS technique is sensitive to the orientation of the adsorbed protein^23^. Therefore, if the orientation of the protein cannot be controlled there will not be a unique spectrum associated with any given protein when using SERS. Additionally, this technique requires a consumable substrate with a limited shelf life^24,25^. Fortunately these complications can be avoided as several therapeutic proteins are provided in formulation at concentrations high enough that the signal enhancement from SERS is not needed. Here we show that characterization of these proteins is possible using spontaneous Raman spectroscopy of free-proteins in solution, provided the sample holder and optical system are well designed. Our approach was to employ the double-pass, confocal optical geometry illustrated in Fig. 1b, with a sample volume of approximately 25 µL. This sample volume is smaller than the U.S. Pharmacopeia recommended excess volume (overfill) of 100 μL for injections supplied in vial form with a label size of 0.5 mL and larger, so it would be possible to operate the system using the overfill volume present in these products^12^.

Alongside the Raman instrument reported here, we have developed a classification algorithm that is capable of classifying a protein that has degraded. While automated classification algorithms have been widely used for spectral classification^26–30^, the challenge here is to develop a classifier that can detect a myriad of degraded protein forms that that the algorithm has never encountered before. This is a specific case of the general One Class Classification problem in chemometrics^31^. In this problem, there is a large set of data in one class, and a limited or non-existent set of data in the ‘other’ class. This problem occurs in a number of domains with examples including anomaly detection in gas turbines^32^ and classification of documents as relevant to a user^33^. One class classification has been applied for Raman spectroscopy based identification of unknown bacterial strains^34^ and the detection of simple mixtures of chlorinated solvents^35^. This work expands the technique to the Raman spectral analysis of complex molecules with a particular focus on therapeutic proteins.

We demonstrate two different approaches to training the classification algorithm using only “good” protein spectra. In both cases the goal is to train the classifier so that it ignores systematic variations associated with the instrument such as shot noise, alignment drift, etc. and instead focuses on spectral features that are correlated with the protein analyte. In the first approach the algorithm is trained using only spectra from high-quality recombinant human growth hormone (rhGH) and in the second approach the training uses both the high-quality rhGH and spectra from three unrelated high-quality proteins of various sizes. The addition of the different protein spectra improves the algorithms ability to identify the protein variants and the algorithm is demonstrated to correctly classify degraded recombinant human growth hormone (rhGH) with 92% sensitivity and 98% specificity for the range of degraded and un-degraded samples used for this work.

## Methods

### Spectra Acquisition

Raman spectra were collected using a purpose built system, shown in Fig. 1c, designed around the low volume sample holder illustrated in Fig. 1b. The sample holder featured a 100 µm thick fused silica sampling window used for both excitation and collection of the Raman signal. A microscope objective was used to focus the excitation light and collect the Raman scattered light. A gold mirror at the back plane of the sample holder, positioned near the focal point of the microscope objective (NA = 0.75, 40x magnification), increased the excitation energy by redirecting the excitation light back through the sample and increased the system’s Raman signal collection by redirecting forward scattered Raman light back towards the microscope objective for collection.

Recognizing that the Raman signal will be collected primarily from the region close to the focus of the microscope objective, the dimensions of the liquid containing well were set at 4 mm wide, 6 mm long, and 0.8 mm deep along the optical path for a total volume of approximately 19 µL. The sample holder did not need to be any larger than this because the increased sample volume would not improve the amount of sample-laser interaction and the walls and mirror in the sample holder were metallic, so they added no background Raman signal. The depth of the sample holder was set to allow the microscope objective to focus light on the back mirror to achieve an increase in excitation and collection as described above, while at the same time moving the excitation spot as far from the fused silica window as possible to reduce the background signal from the window.

To use the holder a 20–25 µL drop of material was added to the well, over filling it slightly, and then the well was capped with the mirror. This prevented the formation of an air bubble between the sample and mirror, resulted in a consistent sample volume and geometry with no meniscus, and effectively sealed the sample preventing it from drying during long spectrum acquisitions. The inverted configuration ensured that neither the sample holder nor the microscope objective needed to move during sample loading or cleaning operations. This allowed the system to be used without re-aligning the optics each time a new sample was presented to the system.

Sample volumes down to 1 μL are possible with capillary based sample holders^36^ but these have an unavoidable overlap of the excitation laser with the capillary walls resulting in a significant background signal due to the capillary material and an attenuation of the excitation light due to scattering or absorption by the capillary material. The impact of the background signal due to the capillary material can be mitigated by choosing a material with a small Raman cross section such as Teflon, but this approach has not been shown to improve the protein limit of detection^37^.

The excitation laser used with this system was a fiber coupled Ondax laser operating at 830 nm (R0-830-PLR-300-MM-1) delivering approximately 100 mW of laser power to the sample. Light was delivered from the laser to collimating optics via a 105 μm core multimode fiber. The collimated light was passed through a Semrock 830 nm MaxLine Laser clean-up filter to remove any amplified spontaneous emission from the laser and any Raman light generated within the delivery fiber. The filtered light was coupled into the optical path of the microscope objective by a Semrock long pass filter operated as a dichroic mirror. Collected light was passed back through the Semrock filter and then through an additional long pass filter to further attenuate Rayleigh scattered excitation light before being coupled into a fiber bundle for delivery to the spectrometer. A diagram of the system is given in Fig. 1c.

Spectra were acquired using an Acton SP-300i grating spectrometer. The use of a mirror in this sample holder does significantly increase the amount of Rayleigh scattered light collected by the system. To prevent the scattered excitation light from saturating the detector the grating in the spectrometer was adjusted to position the scattered excitation light off of the sensor. To generate the spectra presented here the following procedure was used. For each sample of protein solution, 100 spectra were collected with an integration time of 10 s per spectrum. Cosmic ray events were identified in the 10 s spectra and removed. After cosmic ray removal, the individual spectra were scaled to match the median value of pixels 200 to 300 across the 100 spectra set to account for variation in the laser power. This region was chosen because it was beyond the filter turn on region and generally contained no strong spectral peaks. The individual 10 s spectra were then smoothed across wavelength using the Savitzky-Golay filter function (Matlab) with a degree of 11. A representative sample spectrum was created by taking the mean value of the 100 filtered and smoothed spectra at each wavelength, and a noise spectrum was created for each measurement by taking the standard deviation at each wavelength across the sample spectra.

The sample spectrum resulting from this processing contained Raman and fluorescence signal from the protein, sample holder, and the buffer that made up the bulk of the protein solution. A characteristic background spectrum for each protein buffer was created using the process described above but with the appropriate buffer solution rather than a protein sample. The characteristic background spectrum for the rhGH buffer is shown in Fig. 1d with a 10x magnified Raman spectrum of rhGH for comparison. To generate the protein Raman spectra presented in the results section this characteristic background spectrum was subtracted from the sample signal and any residual fluorescence was removed by performing a positive residual style polynomial subtraction as described in ref.^38^. Calibration of the Raman shift was performed using a polystyrene sample with a well-known Raman spectrum.

### Protein material preparation

The proteins used in this experiment were obtained from several suppliers. Somatropin, recombinantly produced human growth hormone (rhGH), was obtained from Sandoz supplied in 10 mM sodium phosphate buffer at a pH of 7.0 and a concentration of 10.7 mg/ml. This material was frozen and stored at −20 °C for shipping, thawed for aliquoting, and the aliquots stored at −80 °C before being thawed for use. The interferon was obtained from RayBiotech (228-10819-3) as a lyophilized powder from a 1 mg/ml solution containing 2.3 mg Sodium phosphate dibasic and 0.55 mg sodium phosphate monobasic buffer and reconstituted in distilled water at a concentration of 3.5 mg/ml. The interferon was used directly after reconstituting to avoid the potential damage of a freeze-thaw cycle. The Immunoglobulin-G (IgG) was obtained from Bethyl Laboratories (P80-105), purified from serum and at a concentration of 1 mg/mL. The IgG was supplied in phosphate buffered saline containing 0.09% sodium azide. Lyophilized insulin powder was obtained from Sigma (I2643) and reconstituted in a 0.01 N HCl solution at a maximum concentration of 11 mg/ml. The proteins examined here were supplied in, or reconstituted into, variants of Phosphate Buffered Saline (PBS) except for insulin which was reconstituted in 0.01 N HCL. Drug product buffers may contain sugars or similar molecules with unique Raman signatures but here we maintained similar buffers where possible to allow for direct comparison of the limits of detection and to avoid buffer exchange.

**Figure 2:**
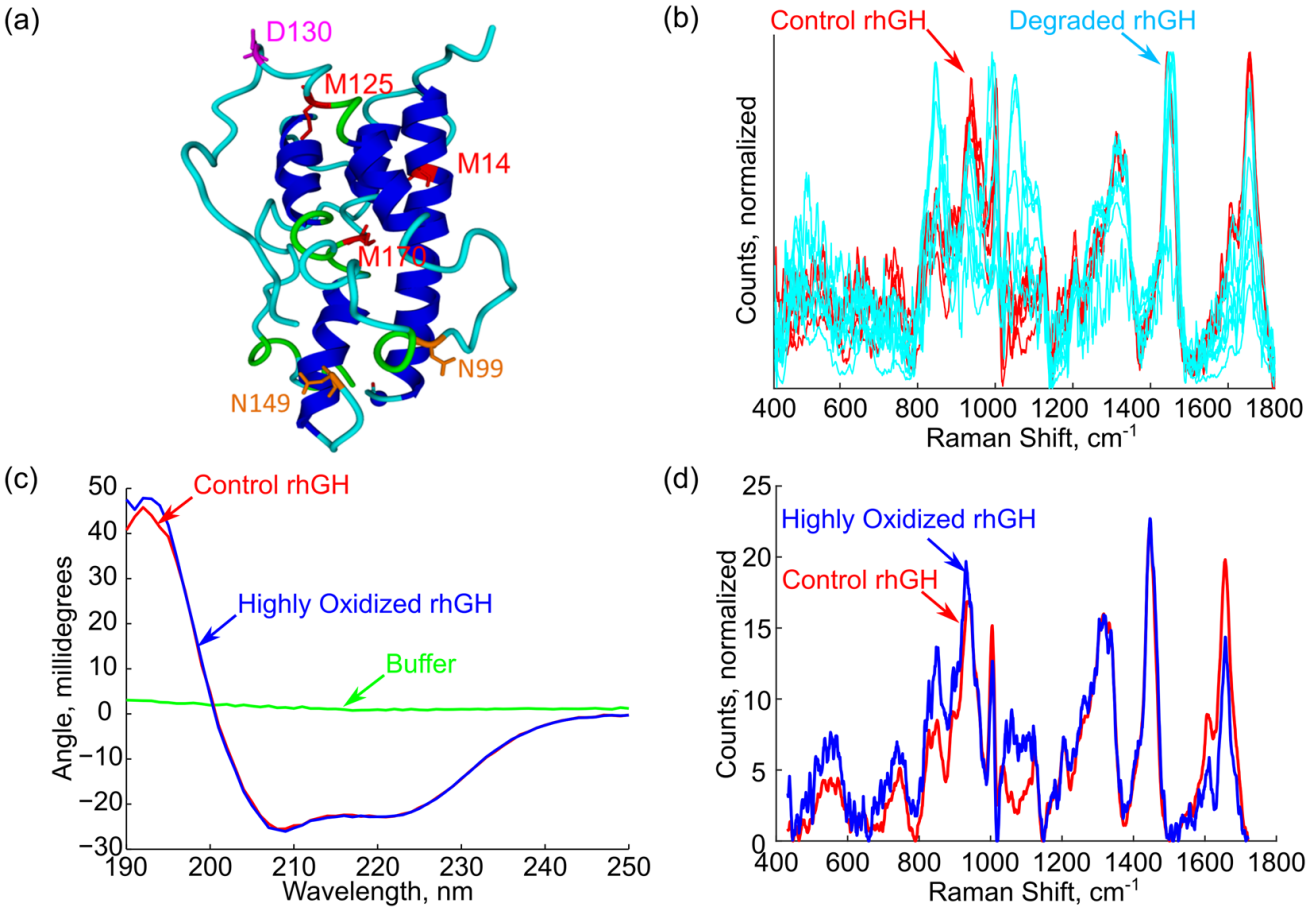
(**a**) Visualization of the sites of potential degradation in human growth hormone. Of particular importance are the methionine residues which each contain a sulphur atom capable of forming a sulfoxide bond. (**b**) Control rhGH in red and degraded rhGH in cyan. The degraded material shows significant, but variable, differences when compared with the control material. (**c**) Circular dichroism spectra for the reference and one sample of highly oxidized human growth hormone, indicating no significant change in the helical portion of the protein under oxidation. (**d**) Raman spectra for the same material in (**c**), presented as counts in 10 s, normalized by concentration.

A series of oxidized human growth hormone samples were prepared from reference material in order to examine the sensitivity of the instrument to changes in proteins due to degradation processes. The oxidized samples were generated by exposing Sandoz Somatropin to 5% hydrogen peroxide (Sigma) and then incubating the material at 37 °C overnight. This resulted in varying levels of oxidation of the Methionine groups M14, M125, and M170. A summary of the oxidation levels of the sample material is given in Table 1, and the oxidation sites are illustrated in Fig. 2(a).

**Table 1:** Oxidation and deamidation summary. This table summarizes the oxidation at methionine sites M14, M125, and M170 and the deamidation at site N149 in the oxidized and stability test samples. The samples were generated and characterized by the Hancock lab at Northeastern University.

	Sequence	Control Samples	Degraded Samples	Oxidized Material With CD Spectra
M14 (ox.)	LFDNAMLR	3 to 14.2%	40 to 100%	90%
M125 (ox.)	DLEEGIQTLMGR	3 to 1.6%	12 to 100%	100%
M170 (ox.)	DMDKVETFLR	5 to 0.1%	3 to 90%	90%
N149 (deamid.)	FDTNSHNDDALLK	0 to 3%	3 to 90%	3%

### Liquid Chromatography - Mass Spectrometry of Oxidized Human Growth Hormone

To prepare the reference and oxidized rhGH samples for LC-MS they were first fragmented into peptides and transferred from their original buffers to a buffer suitable for LC-MS. The first step in fragmenting the proteins was to dissolve the samples in 6 M Guanidinium chloride to denature the proteins. The disulfide bonds within the proteins were then broken by a reduction step using 10 mM dithiothreitol (DTT) at 70 °C for 30 minutes followed by an alkylation step using 55 mM iodoacetamide (IAA) at room temperature for one hour in the dark.

In preparation for digestion, the proteins were then dialyzed three times in Tris-HCl buffer, pH at 6.8, via 10 kD Amicon membrane centrifugal filters (Millipore, Merck, Germany) at 12,000 rpm for 15 minutes. The proteins were then digested with Trypsin at room temperature overnight to fragment the proteins into peptides while minimizing artificial oxidation. The digestion process was terminated with 20 µL 5% formic acid and the peptide solutions were aliquotted to 20 µL samples and stored at −80 °C for further analysis.

A Dionex 3000 UPLC system (Ultimate 3000, Sunnyvale, CA) was used for the liquid chromatographic separation and a Thermo LTQ XL (Linear Ion Trap, Thermo, San Jose, CA) was used for the mass spectrometry. The liquid chromatographic separation used a self-packed capillary column with an internal diameter of 75 μm and a length of 150 mm packed with Magic C18 (3 μm particle, 200 Å pore). The LC separation method was carried out by a 75 minute gradient elution program, using 0.1% formic acid in water as Phase A and 0.1% formic acid in acetonitrile as Phase B, at a flow rate 200 nL/min. The ratio of Phase B was increased from 2% to 40% over the first 50 minutes, then increased to 95% over the next 5 minutes and kept there for 10 minutes. The ratio of Phase B was then decreased from 95% to 2% in 2 minutes and maintained at 2% to the end of the program.

The coupled linear ion trap mass analyzer was operated in the data-dependent CID fragmentation mode and it was adjusted automatically between MS and MS/MS acquisition in nine cycles with a full scan mode at m/z 300-2,000 Da. Thermo Proteome Discover 1.4 was used to analyze the raw data, using a human growth hormone sequence database file and assuming full trypsin specificity. The tolerance for precursor ions was set at 50 ppm and the tolerance for product ions was set at 1.0 Da. Carbamidomethylation of cysteine was selected as a static modification and deamidation on asparagine and oxidation on methionine were selected for dynamic modification search. The final confirmation of the modifications was by manual search.

### Classification of Protein Variants

The goal of this work was to develop a classifier capable of recognizing if a particular sample under test was high-quality rhGH or degraded rhGH. A standard binary classifier could be developed for this problem with an output of either “good material” or “bad material”. The standard classification algorithms for this problem work by defining a boundary between the two classes, based on examples from each class. These algorithms assume that the samples presented to the classifier adequately sample the space of possible spectra generated by each class. However, in this application a complete set of “bad” examples does not exist, the range of degraded products is too large to adequately sample, and instead the classifier must define the boundary with only examples of the “good” class. This problem falls into the general field of one-class classification^39^. A variety of classifiers including Support Vector Machines^40,35^, neural networks^33^, and nearest-neighbors^41^ have been applied to this problem. The approach used in this paper will be to define an ellipsoid in N dimensions (where N is the size of the spectrum for classification), making the classifier presented a variant of the Minimum Volume Ellipsoid approach developed by Rousseuw^42–44^.

Given the high dimensionality of the data (1340 spectral data points in the raw data) and the small number (5) of repeated experiments generated degraded samples overfitting in the classifier is an important concern. Information about protein structure and condition is not uniformly distributed in the spectrum of the protein, so the first step after spectral processing is a dimension reduction to reduce the impact of spectral variation in low information regions on the classification algorithm. After background subtraction and residual polynomial fitting the first step in dimension reduction is to truncate the spectrum to an appropriate spectral region. The region from 800 to 1721 cm^−1^ was chosen because it contains the brightest features associated with protein structure and composition, resulting in a sample spectrum represented by a vector 830 points long. The data is then scaled into the region 0-1, to reduce the impact of small changes in concentration. The next step is to apply Principal Component Analysis (PCA) to further reduce the dimensionality of the data. The PCA algorithm finds a set of vectors in the original space that can be used to represent the data. The vectors are ranked by the amount of variation in the original data each vector captures. By using a set of principal components smaller than the dimension of the original space, the length of the vector representing each data point is reduced. Additionally, if the data set for the PCA algorithm is chosen correctly then the principal components themselves should be directly related to spectral information relevant to classifying the proteins.

Two methods of performing the initial principal component analysis for dimensionality reduction were investigated. In Method 1 the PCA algorithm was applied to a one set of spectra of the high-quality (un-degraded) rhGH. In Method 2 the spectra for the high-quality rhGH were combined with spectra from the three other high-quality proteins. The high-quality rhGH training set was formed from five data sets collected over the course of the year. As part of this research the impact of the number of data sets included in the training set was investigated. In both Method 1 and Method 2, the N-highest ranked principal components were used to form a reduced dimensional space, and the test data was projected into this space. N was chosen such that the resulting PC space captured at least 60% of the variation in the training set. This reduced dimension data set was used as the input to the classifier.

The classifier examined here is constructed by defining an ellipsoidal surface that encloses the spectra representing the high-quality rhGH material. The ellipsoid is chosen to follow the shape of the high-quality rhGH material and the resulting classifier can be written from the equation of an ellipsoid in N dimensional space as

1 $$f({\bf{x}})={(\frac{{({\rm{A}}{\bf{x}}-{\bf{c}})}_{1}}{{\sigma }_{1}})}^{2}+{(\frac{{({\rm{A}}{\bf{x}}-{\bf{c}})}_{2}}{{\sigma }_{2}})}^{2}+\ldots +{(\frac{{({\rm{A}}{\bf{x}}-{\bf{c}})}_{N}}{{\sigma }_{N}})}^{2}={|({\rm{A}}{\bf{x}}-{\bf{c}})\oslash {\boldsymbol{\sigma }}|}^{2}\le {\rm{\Theta }}$$

where **x** = (*x*_1_, …, *x*_*N*_) is the representation of the spectrum in the N dimensional space generated after the first round of PCA, **σ** is the vector representing the ellipse axes lengths, and Θ is a tuning parameter which controls the size of the ellipse. ⊘ is the Hadamard division operator indicating element-wise division of two vectors. The term (A**x** − **c**) uses a projection matrix A and centroid vector **c** to project the data from the N-dimensional PC-projected space into an N-dimensional space centered on the classification ellipsoid with its coordinate axes aligned with the ellipsoids axes. A second round of PCA is performed using just the rhGH training data to define the matrix A, centroid vector **c**, and axis length vector **σ**. In this round the principal components now form the axes of the ellipse and set the projection matrix, A, the eigenvalues are used to set the relative size of the ellipse along each axis, **σ**, and the centroid of the projected high-quality rhGH spectra, **c**, is used to shift the projected data into the center of the ellipse. In this way the longest dimension of the ellipse will be aligned with the direction in which the un-degraded rhGH spectra are most spread, and the shortest dimension will be aligned with the direction in which the data are least spread. The performance of this classifier depends on the value of Θ. As a sample independent approach, the value of Θ can be set so that the classification ellipsoid captures 99% of the training examples.

## Results

The characteristic spectrum of four proteins is shown in Fig. 1e. Each spectrum in the figure represents one 1,000 second measurement (100 × 10 s spectra as discussed previously). These spectra are consistent with those reported in previous work at high concentrations and larger sample volumes^16,45–47^. A detailed peak assignment and interpretation is given in the supplementary material. To gauge the repeatability of the system several spectra were taken of rhGH over a long time period at a fixed concentration and of Insulin over a short time period with a variable concentration as indicated in Fig. 1e.

### Protein Oxidation

Oxidized human growth hormone was prepared and analyzed by mass spectrometry, circular dichroism spectroscopy, and by Raman spectroscopy. A summary of the mass spectrometry analysis is given in Table 1, the Circular Dichroism result in Fig. 2c, and the Raman spectra of the highly oxidized protein is presented in Fig. 2d.

Figure 2b shows the Raman spectra of the degraded material along with the spectra of the control material. The spectra of the degraded material show large changes in the Raman response. To verify that there were no gross changes in the structure of the protein a set of circular dichroism spectra were taken of the most highly oxidized material and control material using a Circular Dichroism Spectrometer (Aviv Model 202). The CD spectra, normalized by sample concentration as measured using UV-Vis absorption (NanoDrop A280), are shown in Fig. 2c. Circular Dichroism spectrum of the control and oxidized human growth hormone are consistent with an α-helix protein structure. A change in the circular dichroism spectrum of the protein would be expected if the helices of the protein were unfolding due to the oxidation process, but the experimental spectra show no change between the highly oxidized and control material. This indicates that there is no major change in the α-helix segments in the rhGH. However, human growth hormone is only about 45% α-helix^48^. The changes in the Raman spectra may be due to structural changes in the non-helix regions of the rhGH in addition to changes due to the chemical process of oxidizing the Methionine residues. Currently the changes in the Raman spectra do not result in a quantifiable measurement of protein oxidation, the changes are too large and too variable to be directly tied to protein oxidation. Rather than trying to produce a quantifiable measure of oxidation an alternate approach was pursued, developing a classifier to give a binary measure of the protein as either “good” or “bad”.

### Protein Variant Classification

Two variations of the classification scheme outlined in the methods section were examined: one which uses only spectra from rhGH to define the classification boundary (Method 1) and one which also includes spectra from independent high quality proteins in the first step of dimension reduction (Method 2). In both approaches there are three parameters that can be adjusted to control the performance of the classifier: the number of rhGH spectra included in the first round of PCA, the size of the first PC space, and the relative size of the classification ellipse which is controlled by the tuning parameter Θ. The number of rhGH spectra included in the first round of PCA was kept variable to examine how the performance of the classifier changed as the amount of information about the high-quality rhGH increased while the other two parameters were set as discussed below.

The size of the first PC space can be set by making a decision about how much variation in the input data should be captured in the PCA. The eigenvectors associated with each principal component can be used as a proxy for the amount of variation in the original training data captured by each principal component. The higher this parameter is set, the more principal components will be required to represent the data. Aiming to capture ~60% of the variation in the first input set results in a PC space of 9–30 components, representing a significant reduction in dimensionality while maintaining most of the variation in the data. The size of the classification ellipse was set so that the classifier identified 99% of the test data set as “good” material using the assumption that the classifier should at least be able to identify the training set as “good” material.

**Figure 3:**
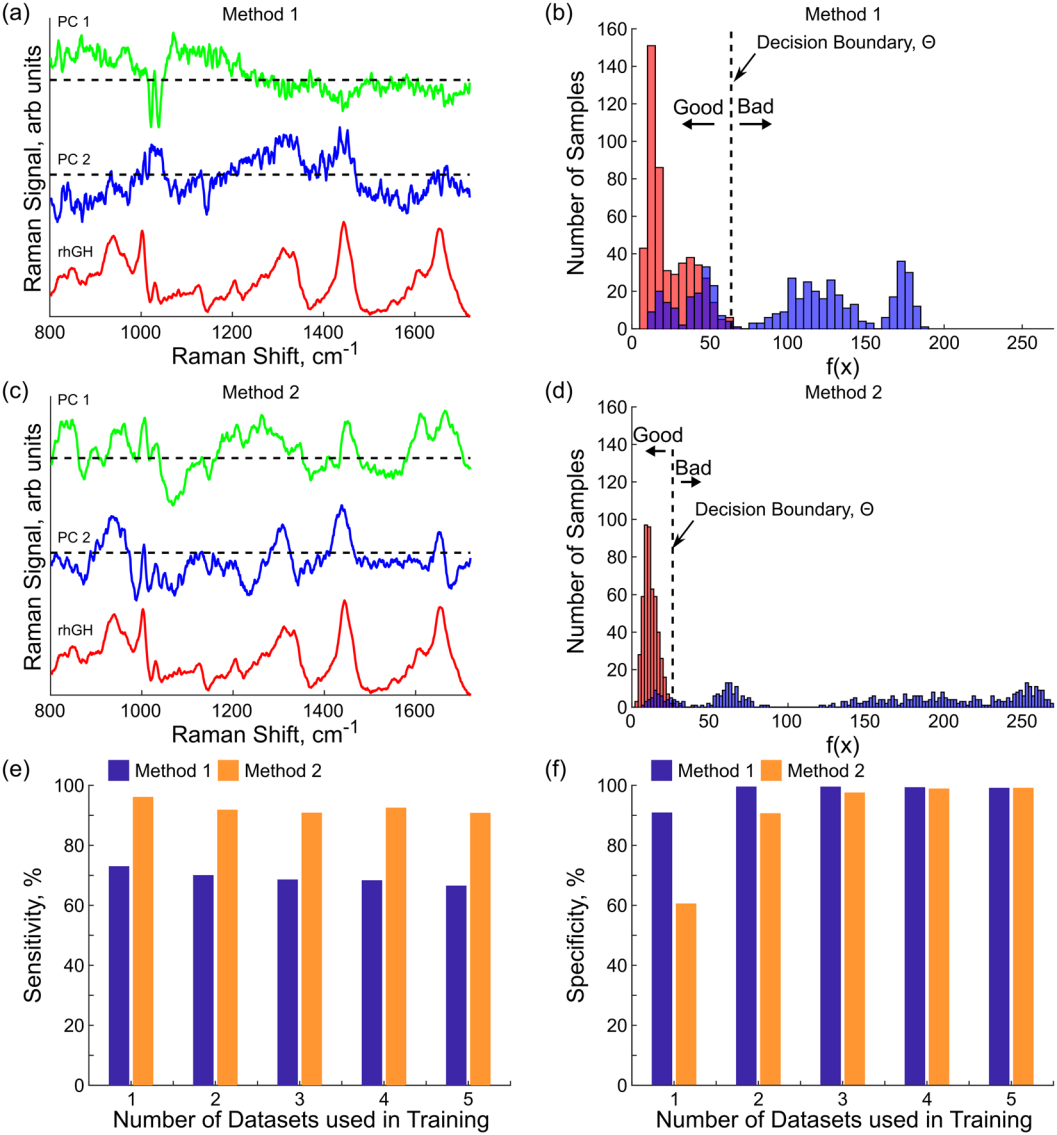
First principal components along with rhGH spectra for method 1, use only rhGH spectra, in (**a**) and for method 2, use spectra from all four proteins in (**c**). Histograms of the good and degraded materials along with classification boundary are shown in (**b**) for method 1, and in (**d**) for method 2. The red bars represent spectra from the reference high quality rhGH and the blue bars represent spectra from the degraded proteins. The “good” and “bad” labels indicate the decision of the classifier. Sensitivity (**e**) and selectivity (**f**) are shown for both methods as a function of the number of datasets used in training the classifier. This is with the algorithm set to capture 60% of the variation in the first round of PCA and 99% of the training data when setting the tuning parameter, Θ. As the size of the training set becomes larger, the classification ellipse must also become larger to continue capturing 99% of the training set. This generally results in an improvement in the specificity, but a decrease in the sensitivity of the classifier.

With N set to capture 60% of the training data variation and Θ set so that the classification ellipse encloses 99% of the training set, it is possible to compare the results of the two methods of generating the first principal component space. Figure 3 shows the principal components that result from performing the first round of PCA on either just rhGH data (3a), or rhGH data along with additional non-rhGH data (3c), along with a spectrum of rhGH for comparison. While both methods produce principal components with some peaks at locations associated with features in the proteins, the principal components produced when using information from additional proteins show more clearly defined features around regions that have been identified as having information about protein structure and composition.

As expressed in Equation 1, the classifier reduces the entire spectrum to one number, f(x), and then compares that number with a threshold value. Figure 3b,d show the histograms of f(x) for the high-quality (red) and degraded (blue) samples under both methods for the case when the training rhGH set contains three of the five high-quality rhGH data sets. The dashed line in both figures indicates the decision threshold value Θ, with material to the left of the dashed line identified as “good” and the material to the right of the line identified as “bad”. Under both methods the majority of the “good” reference material is identified as good because the value of Θ was chosen to pass 99% of the training set and because the variability among the good rhGH data sets was small. On the other hand, from the histograms it’s clear that the variability of the degraded material is fairly large, with some fraction appearing as quite similar to the “good” material and some appearing as quite distinct. In fact, from the histograms it appears that the degraded material comes from a few discrete sets of possible degraded material justifying our assumption that we did not fully sample the space of possible degraded material.

To explore the performance of the classifier as the amount of information available increases the number of training examples to use in the first round of PCA was varied, using one, two, three, four, or all five sets of spectra collected over the course of a year. The bar charts in Fig. 3 shows how the sensitivity (3e) and specificity (3 f) vary as the number of spectra used during the first round of PCA increases for both Method 1 and Method 2. The reported sensitivity and specificities are the average across all possible combinations of spectra (5 combinations for using one or four sets of data, 10 for using two or three sets of data, and 1 for using all five sets of data). The specificity of the algorithm, its ability to identify non-degraded rhGH as “good”, improves as the number of data sets used to train the algorithm increases since the algorithm is set to capture 99% of the training data when setting the tuning parameter Θ. Since capturing more of the data requires a larger ellipsoid, the sensitivity of the algorithm in identifying degraded material as degraded decreases as the set of training data becomes larger. This is partially the result of some of the degraded material having spectra very similar to the non-degraded material. A good balance for this data set is obtained by using three of the five datasets in the training process. The sensitivity of the classifier, its ability to correctly identify degraded material, is significantly improved when the first round of PCA incorporates non-rhGH proteins. This is likely because of the fact that principal components are chosen to be vectors that represent change in the set of spectra used to generate them. In Method 1, training with just rhGH, the highest ranked principal component matches the feature that changes the most among the set of “good” rhGH. Since all of the inputs are “good” rhGH this vector shouldn’t map to a physical feature in the protein since that feature should be common to all of the samples. In Method 2, training with rhGH and other proteins, to describe the variation among the proteins the principal components must describe the features in the protein that change between the different protein samples. In this way the inclusion of other proteins should bias the first step of the algorithm to generate principal components that have strong features at positions associated with protein structure since the set of spectra used to generate the principal components has significant variation in this features.

## Discussion

The classification system discussed here can provide a binary judgment on the quality of a protein drug product without requiring the operator to interpret the spectrum of the protein. From the point of view of the end user they place a small drop of material into the system and get a “probably good” or “probably bad” indication. This basic approach allows such a system to be deployed in a point of care application since it does not require any expertise on the part of the user to evaluate the data, and only requires the ability to dispense a small sample of drug product. Physically, the current system takes the form of a large bench-top instrument but it is possible to reduce the size to something approaching a shoebox allowing deployment beyond clinical locations. As developed here, the system does have some limitations.

Currently the system relies on example spectra from “good” proteins at the correct concentration to form its classification boundary and can separate the “good” from “bad” proteins with reasonable accuracy on the set of good and degraded proteins examined here. Due to the pre-processing scaling of the Raman spectra into the 0–1 range the classifier is insensitive to small changes in protein concentration. However, a change in concentration can signal that the drug product being tested is not the correct formulation or has undergone some degradation process. Monitoring the scale factor or testing using a UV absorption measurement to verify the protein concentration should be done as part of the drug quality check. The algorithm presented builds a model for “good” proteins based on examples of the good protein. This must be done separately for each drug product to be evaluated by the system, and then in use the system must be told what drug it is examining. Finally, the data presented here represent a fairly small set of both high quality and degraded drug product. In terms of oxidation, summarized in Table 1, the control material had up to 14% oxidation at methionine 14, while the degraded material samples start at 40% oxidation at that methionine residue. The results from this data are promising, but further work will be required to verify that the approach works across a larger range of control and degraded material and to examine how the algorithm responds to smaller changes in the protein.

## Conclusions

Characterization of proteins at or below their therapeutic dose concentration within the correct buffer has numerous applications ranging from process analytics during the manufacture of bio-therapeutics to point of care applications where the quality of a protein can be checked before being administered to a patient. The system presented here can make repeatable spectral measurements of proteins at relevant concentrations and the classification algorithm can be used to identify “good” proteins without having to sample the entire space of “bad” proteins. Point-of-care applications benefit from the small sample volumes (25 μL) utilized for these experiments, which is small enough that it could be taken from the “overfill volume” used to ensure that a syringe can withdraw a full dose from a vial^12^. This approach allows testing of both the batch and single dose of the drug even in cases when the cost of sacrificing even a single dose is prohibitive, and will allow a hospital or clinic to identify drugs that have degraded unexpectedly or to find counterfeit drugs which have slipped into the supply chain. By monitoring every dose, they will be able to catch problems that they would have missed when only checking batches of medicine they suspect are degraded or counterfeit.

Stepping beyond therapeutic protein products, the system described here could be used to verify the integrity of vaccines. As stated in the introduction, during one study of the vaccine distribution cold chain in India as much as 76% of the vaccine vials tested showed evidence of spoilage^11^. A system similar to the one presented here could be used to verify the quality of the vaccine material being used during a vaccination event. By catching spoiled material before it’s administered to patients the efficiency of a vaccination program can be improved. A more aggressive application of the system may allow a reduction in vaccine wastage. A large source of wastage in vaccine delivery in India has to do with the incomplete utilization of vaccines packaged in multi-dose vials^8^. When a vaccination event is held the best practices require that at the end of the event any opened vaccine vial be discarded, and that any unopened vial that has been issued but unused across three vaccination sessions also be discarded^8^. This is to ensure the safety and efficacy of the vaccines. A system that can determine if the vaccine in an opened vial is still good could allow the re-issuing of opened vaccine vials and unused but previously issued vials, while at the same time catching material that should be in good condition but that may have experienced degradation due to an undocumented problem with storage conditions. In this application there is a direct risk to the patient, since it will allow the use of material that would have previously been discarded, and determining if the system presented here is adequate for this task will require extensive testing on a wide range of high quality and degraded vaccine material. Beyond protein based medicine and vaccines, there is currently need for a counterfeit medicine detection tool. The World Health Organization Global Surveillance and Monitoring Systems report^49^ estimates that about US$30.5 billion is spent worldwide on substandard and falsified medical products, resulting in significant threats to the health of patients.

The basic approach presented in this paper, build a classifier based on the normal state of a protein and then try to determine when that protein is degraded, can also be applied to diagnostic applications of Raman spectroscopy. Particularly, this approach can be applied to the analysis of biofluids which is an active area of spectroscopy research^50,51^. Changes in the gylcosylation state of mucin proteins in mucus/sputum could be used for diagnosis of COPD^52^, levels of serum albumin can be indicative of liver failure^53^, and relative levels of albumin to IgG, IgA, and IgM can be markers for diseases of the kidneys and liver^54^. Mucin proteins in mucus^55^ along with albumin, IgG, IgA and IgM in serum^56^ all occur at concentrations high enough to be directly visible in the system presented here. In this diagnostic application there can be a wealth of data for the “no-disease” state from the population of healthy patients and a much smaller amount of data from the population with a disease. A variant detection approach could be used to identify that a change has occurred in the patients’ serum or mucus proteome, signaling the need for further investigation.

## Electronic supplementary material

Supplementary Information
