# Supplementary material for: Optical Detection of Degraded Therapeutic Proteins
Source: Sci Rep. 2018 Mar 23;8:5089. doi: 10.1038/s41598-018-23409-z (PMC5865131; doi:10.1038/s41598-018-23409-z)
Supplement: Supplementary file 1 — Supplementary Information [file 41598_2018_23409_MOESM1_ESM.doc]

**Optical Detection of Degraded Therapeutic Proteins**

William F. Herrington Jr.*†, Gajendra P. Singh†, Di Wu‡, Paul W. Barone†, William Hancock‡, Rajeev J. Ram†

† Massachusetts Institute of Technology, Cambridge, Massachusetts 02139, United States

‡ Northeastern University, Boston, Massachusetts 02115, United States

**Supplementary Information**

*Spectra assignments and interpretation*

The peak assignments in Figure S1 and Table S1 were based on the results in references [ 1, 2, 3, 4, 5] and comparison among the proteins. Features in the Raman spectra of the proteins were associated with the protein backbone, secondary structure, and amino acid side chains. All four proteins produced spectra with a strong Amide I band around 1660cm^-1^ due to the C=O bond in the protein backbone, and most exhibited some peaks associated with the Amide III band. Also common to the spectra were peaks associated with CH2 deformation, CH, C-C, and C-N modes. The remaining identified strong peaks were associated with Tyrosine and Phenylalanine. Along with the identified peaks, there were generally one or two strong features that we were unable to assign based on the prior literature.

For interferon, insulin, human growth hormone, there are small variations between the position of the spectral peaks identified here and their position in prior work. Given the sensitivity of the peak positions to the condition of the proteins and pH of the buffer this is not particularly surprising. There are greater differences between the peaks identified in the IgG samples in this work when compared to prior work, but IgG itself is expected to be variable in composition.


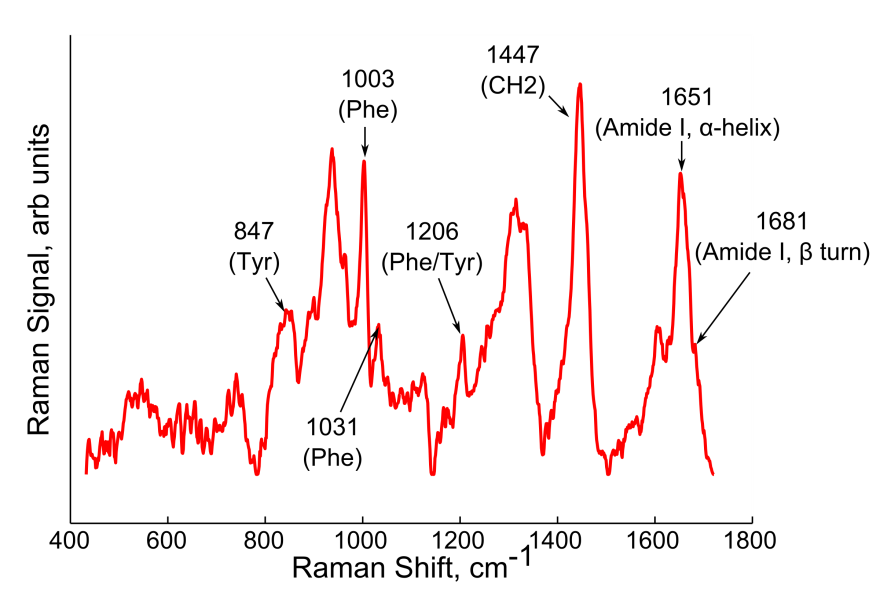


**Figure S1** Peak assignments for the Somatropin (rhGH) obtained from Sandoz.

**Table S1** This table lists the peaks present in each protein measured.

| **Peak** | **Insulin** | **Interferon** | **rhGH** | **IgG** |
| --- | --- | --- | --- | --- |
| **Amide I** | 1665 | 1658 | 1651 | 1666 |
| **Amide III** | 1246-1265 | 1314-1331 | - | 1236 |
| **C-C or C-N** | 943, 1124 | 1124 | - | 1072 |
| **CH2** | 1448 | 1433 | 1447 | 872, 1447 |
| **CH deformation** | 1336 | - | - | - |
| **Phenylalanine** | 647,1005,1032, 1207,1611 | 1005,1028, 1207 | 1003, 1031, 1206 | 1010, 1039 |
| **Tyrosine** | 664, 833-849, 1181, 1207, 1611, 1619 | 836-847  1196-1207 | 847, 1206 | 751 |
| **Unidentified strong peaks** | 890 | 920, 934 | 937, 1302-1337 (Amide III?) | 988,  1338 (Amide III?) |

The peak assignments were made by comparison with the literature: insulin peaks from [ 3], interferon from [ 1], rhGH from [ 2], and IgG from [ 4]. In addition to the direct comparison with previous protein spectra, assignments were also made based on comparison with the amino acid Raman spectra [ 5] and among the proteins investigated within this work. In addition to the peaks listed, the insulin spectra exhibit a peak at 520 cm-1 associated with S-S vibrations and a peak at 741 cm-1 that has been assigned to skeletal bending in the literature.

*Protein Spectra at limiting concentrations*

For reliable product identification, the system should be capable of measuring the spectra below the dose concentration. As seen in Figure S2, and summarized in Table 1, the system can measure the protein spectra down to concentrations below the dose concentration for insulin, rhGH, and IgG. While the performance of the system when measuring interferon is similar to that of other proteins the dose concentration of interferon is small enough that this system would not be able to perform product identification at the dose concentration.

To be useful in a point of care application the system must be able to accurately measure the Raman spectra of the drug products at dose relevant concentrations. For each of the four proteins examined here a series of spectral measurements were made across a range of concentrations. The highest concentration measured varied between 10 mg/mL for rhGH and 1mg/mL for IgG. The lowest concentration measurements were carried out on samples at below 0.5 mg/mL. The spectra for the high concentration and lowest concentration consistent with the high concentration are given in Figure 4 for the four proteins of interest.


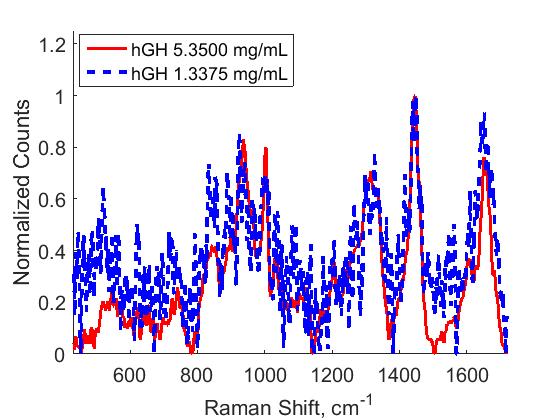

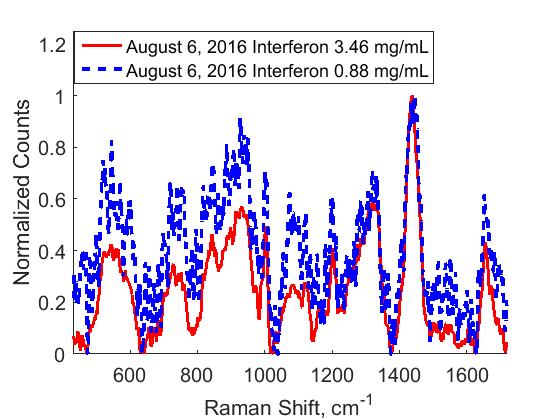


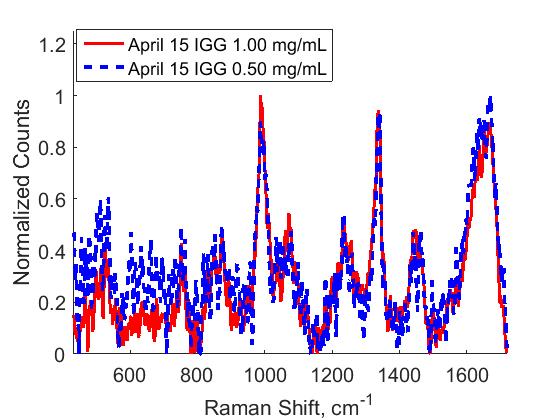

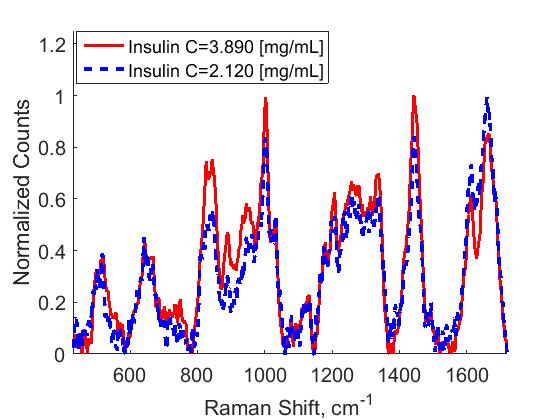


**Figure S2** Raman spectra of (a) Insulin, (b) Interferon, (c) rhGH, and (d) IgG at high and low concentrations. These spectra are to the brightest peak after background subtraction. The spectra at low concentrations show more noise than the high concentration spectra but are still representative of the Raman spectra of the proteins.

**Table S2**: Therapeutic products, concentrations and limits of detection

|  | **Size (kDa)** | **Dose Concentration** | **Lowest Reported**  **Measurement**  **Previous Work** | **Lowest usable**  **Concentration**  **1000s integration**  **This Work** |
| --- | --- | --- | --- | --- |
| **Insulin** | 5.8 | Typical  3.46 mg/mL (100U)  Range  0.87-17.35 mg/mL | 0.581 mg/mL [ 6]  633nm 10-30mW excitation,  Drop Coating Deposition Raman | 2.1 mg/mL |
| **IFNa2b** | 19.2 | IntronA  0.038 -0.192 mg/mL | 50 mg/mL [ 1 ]  514.5nm 500mW excitation,  Standard Raman | 0.880 mg/mL |
| **rhGH** | 22.1 | Genotropin  0.8 – 12 mg/mL  (0.6-36 U) | 8 mg/mL [ 2]  244nm 26mW excitation  UV Resonance Raman | 1.34 mg/mL |
| **IgG** | 143.8 | 10 mg/mL | 1 mg/mL [ 7]  830nm 60mW excitation  Standard Raman | 0.5 mg/mL |

**Table S3:** Average performance of classification algorithm for different training set sizes.

|  | **Use one set** | **Use two sets** | **Use three sets** | **Use four sets** | **Use five sets** |
| --- | --- | --- | --- | --- | --- |
| **M1, 60%**  **Sensitivity** | 72.85 | 69.89 | 68.43 | 68.17 | 66.38 |
| **M1, 60%**  **Specificity** | 90.76 | 99.42 | 99.40 | 99.20 | 99.00 |
| **M2, 60%**  **Sensitivity** | 95.96 | 91.70 | 90.70 | 92.38 | 90.64 |
| **M2, 60%**  **Specificity** | 60.44 | 90.52 | 97.44 | 98.76 | 99.00 |

Note: This is with the algorithm set to capture 60% of the variation in the first round of PCA and 99% of the training data when setting the tuning parameter, Θ. As the size of the training set becomes larger, the classification ellipse must also become larger to continue capturing 99% of the training set. This generally results in an improvement in the specificity, but a decrease in the sensitivity of the classifier.

# References

x

| 1. | Williams, R. W., Secondary structure of human leukocyte interferon from Raman spectroscopy. *The Journal of Biological Chemistry* **260** (7), 3937-3940 (1985). |
| --- | --- |
| 2. | Mulinacci, F., Bell, S. E. J., Capelle, M. A. H., Gurny, R. & Arvinte, T., Oxidized Recombinant Human Growth Hormone That Maintains Conformational Integrity. *Journal of Pharmaceutical Sciences* **100** (1), 110-122 (2011). |
| 3. | Manjialardo, S., Piccirilli, F., Perucchi, A., Dore, P. & Postorino, P., Raman analysis of insulin denaturation induced by high-pressure and thermal treatments. *Journal of Raman Spectroscopy* **43** (6), 692-700 (2012). |
| 4. | Kengne-Momo, R. P. *et al.*, Protein Interactions Investigated by the Raman Spectroscopy for Biosensor Applications. *International Journal of Spectroscopy* **2012** (2012). |
| 5. | Zhu, G., Zhu, X., Fan, Q. & Wan, X., Raman spectra of amino acids and their aqueous solutions. *Spectrochimica Acta Part A: Molecular and Biomolecular Spectroscopy* **78**, 1187-1195 (2011). |
| 6. | Drachev, V. P., Thoreson, M. D., Khaliullin, E. N., Davisson, V. J. & Shalaev, V. M., Surface-Enhanced Raman Difference between Human Insulin and Insulin Lispro Detected with Adaptive Nanostructures. *The Journal of Physical Chemistry B* **108** (46), 18046-18052 (2004). |
| 7. | Paidi, S. K. *et al.*, Rapid Identification of Biotherapeutics with Label-Free Raman Spectroscopy. *Analytical Chemistry* **88** (8), 4361-4368 (2016). |

x
